# Gene expression responses of tomato inoculated with *Pectobacterium carotovorum* subsp. *carotovorum*

**DOI:** 10.1002/mbo3.911

**Published:** 2019-09-19

**Authors:** Arnaud T. Djami‐Tchatchou, Lerato B. T. Matsaunyane, Khayalethu Ntushelo

**Affiliations:** ^1^ Department of Agriculture and Animal Health, Science Campus University of South Africa Florida South Africa; ^2^ Agricultural Research Council‐Vegetable and Ornamental Plants Tshwane South Africa; ^3^ Present address: Department of Biology Washington University St. Louis MO USA

**Keywords:** defense, gene expression, *Pectobacterium carotovorum* subsp. *carotovorum*, tomato

## Abstract

Defense responses of tomato (*Solanum lycopersicum* L.) against attack by *Pectobacterium carotovorum* subsp. *carotovorum* (*Pcc*), the causal agent of soft rot diseases, were studied. The expression of some tomato defense genes were evaluated by real‐time PCR quantification analysis, 24 and 72 hr after actively growing tomato plants were inoculated with *Pcc*. These included: *MYB transcriptor factor*, *ethylene response element‐binding protein*, *suppressor of the G2 allele of Skp1*, *cytochrome P450*, *small Sar1 GTPase*, *hydroxycinnamoyl‐CoA:quinate hydroxycinnamoyl transferase*, *pathogenesis‐related protein 1a*, endo‐1,3‐beta‐glucanase, chitinase, proteinase inhibitor, *defensin*, CC‐NBS‐LRR resistance protein, and *phenylalanine ammonia lyase*. The results showed dynamic transcriptomic changes, with transcripts exhibiting different expression kinetics at 24 and 72 hr to confer resistance to tomato against *Pcc* infection.

## Graphical abstract

Targeting various disease response‐related genes, real‐time PCR was performed on tomato leaf tissue sampled 24 and 72 hr postinoculation with *Pectobacterium carotovorum* subsp. *carotovorum* (*Pcc*). The results showed dynamic transcriptomic changes, with transcripts exhibiting different expression kinetics at 24 and 72 hr to confer resistance to tomato against *Pcc* infection.

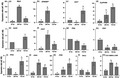

## 1 INTRODUCTION

Tomato is a host of *Pectobacterium carotovorum* (*Pc*), the cause of bacterial soft rot disease (Rosskopf & Hong, 2016). During infection, plants generally respond by activating broad‐spectrum defense responses both locally and systemically in addition to their basal resistance (Djami‐Tchatchou, Allie, & Straker, 2013; Jones & Dangl, 2006). This leads to the induction of pathogenesis‐related (PR) proteins which are indicators of plant‐induced defense responses (Mitsuhara et al., 2008). However, specific responses by plants to attack by specific pathogens are far from being completely elucidated. Among the unknown responses are the expressions of *MYB transcriptor factor*, e*thylene response element‐binding protein*, s*uppressor of the G2 allele of Skp1*, *cytochrome P450*, *small Sar1 GTPase*, *hydroxycinnamoyl‐CoA:quinate hydroxycinnamoyl transferase*, *pathogenesis‐related protein 1a*, endo‐1,3‐beta‐glucanase, chitinase, proteinase inhibitor, *defensin*, CC‐NBS‐LRR resistance protein, and *phenylalanine ammonia lyase* when the tomato plant is attacked by *P. carotovorum* subsp*.* *carotovorum *(*Pcc*). The main objective of this study was therefore to investigate the effects of inoculation of tomato leaves with *Pcc* on tomato defense responses based on the relative expression of selected genes.

## 2 MATERIALS AND METHODS

Three‐week‐old seedlings of tomato, cultivar Heinz 1370, grown in the glasshouse under overhead irrigation at a 25/15°C day/night regime were selected for the study. The plants were inoculated with the bacterial strain BD163 of *P. carotovorum* subsp. *carotovorum* (*Pcc*) (10^5^–10^8^ CFU/ml) suspended in inoculation buffer (0.0014 M KH_2_PO_4_, 0.0025 M Na_2_HP0_4_, pH 7.00), and control plants were mock inoculated with the buffer. *Pectobacterium* cells suspended in the inoculation buffer were pressure infiltrated (Djami‐Tchatchou, Maake, Piater, & Dubery, 2015) at the base of the underside of a tomato leaf. In total, 15 plants were included in the study. Four whole leaves were infiltrated per plant.

The study consisted of five treatments. Four sets of three plants were either inoculated with buffer or with the bacterium and harvested after 24 and 72 hr. A final negative control was also included, with leaves from three untreated plants harvested at the beginning of the experiment. Once harvested, all leaves were kept at −80°C and then the frozen samples were ground in liquid nitrogen using a mortar and pestle.

Total RNA was extracted from 100 mg ground leaf tissue using the TRIzol® Reagent method (Invitrogen). The extracted RNA was treated with DNase I (Thermo Scientific). From the total RNA, cDNA was synthesized using a RevertAid™ Premium First Strand cDNA synthesis kit (Fermentas, Thermo Scientific). For accurate calculation of relative gene expression by qPCR, the input RNA was standardized to the same single concentration for cDNA synthesis across all treated and untreated samples.

During the cDNA synthesis, control reactions lacking reverse transcriptase (RT), referred to as a non‐RT control, were set up to check the samples for DNA contamination. The non‐RT contained the same amount of total RNA as the experimental samples, with the oligodT primer, dNTPs, reaction buffer, ribolock except RT. The products from the cDNA synthesis reactions were used for the qPCR and all the control reactions lacking RT samples exhibited no DNA contamination by showing no amplification. Quantitative real‐time PCR was performed using a rotor Gene‐3000A instrument (Qiagen) and the SensiFAST SYBR No‐ROX Kit (Bioline) according to the manufacturer's instructions to quantify the expression of defense‐related genes *MYB transcription factor*, *ethylene response element‐binding protein* (EREBP), *suppressor of the G2 allele of Skp1* (SGT1), *cytochrome P450*, *small Sar1 GTPase* (SAR1‐GTPase), *hydroxycinnamoyl‐CoA:quinate hydroxycinnamoyl transferase* (HQT), *pathogenesis‐related protein 1a* (PR1), endo‐1,3‐beta‐glucanase (PR2), chitinase (PR3), proteinase inhibitor (PR6), *Defensin* (PR12), CC‐NBS‐LRR resistance protein, and *phenylalanine ammonia lyase* (PAL). Information on the primers used appears on Appendix Table A1. The experiment was performed using three biological replicates (repeats) in plants. One biological replicate was a pool of four inoculated leaves from different plants of a single genotype. This approach was recommended by Brady et al. (2015) because independent replication is the foundation of any successful hypothesis test; instead of repeatedly sampling the same individual, it is better to sample multiple individuals to minimize the potential for bias in the analysis. The melting curve of the qPCR, always showed a single peak confirming that a single amplicon had been generated by qPCR with no other unspecific amplification which could be from a contaminating genomic DNA. Quantification of the relative changes in gene expression was performed using the relative standard curve method (Djami‐Tchatchou et al., 2015; Djami‐Tchatchou, Ncube, Steenkamp, & Dubery, 2017) with *elongation factor 1‐alpha* and* actin* *8* used as references genes. Datasets were statistically compared between non‐treated samples and treated samples at each time point using one‐way analysis of variation with the statistical analysis software GraphPad inStat 3 (GraphPad Software). The confidence level of all analyses was set at 95%, and values with *p* < .05 were considered significant.

## 3 RESULTS AND DISCUSSION

**Figure 1:**
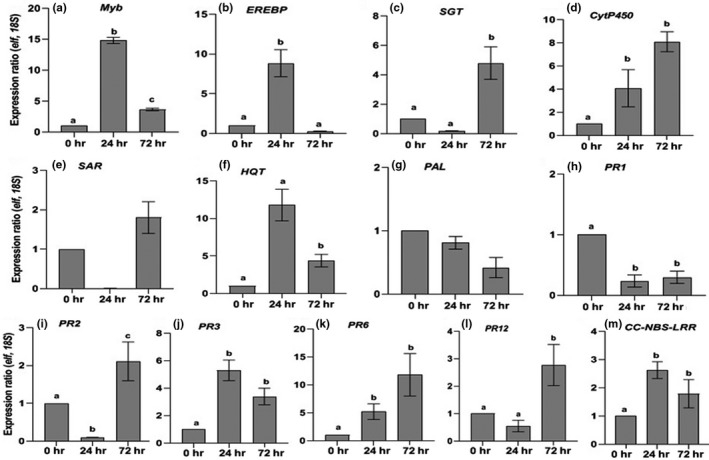
Defense‐related genes expression analysis in tomato following *Pectobacterium carotovorum* inoculation. (a) *MYB transcriptor factor,* (b) *Ethylene response element‐binding protein* (ERBP), (c) *Suppressor of the G2 allele of Skp1* (SGT1), (d) *Cytochrome P450*, (e) *Small Sar1 GTPase* (SAR1‐GTPase), (f) *Hydroxycinnamoyl‐CoA:quinate hydroxycinnamoyl transferase* (HQT), (g) *Phenylalanine ammonia lyase* (PAL), (h) *Pathogenesis‐Related protein 1a* (PR1),(i) Endo‐1,3‐beta‐glucanase (PR2), (j) Chitinase (PR3), (k) Proteinase inhibitor (PR6), (l) *Defensin*(PR12) and (m) Cc‐nbs‐lrr resistance protein. The data was normalized using *Elf α* and *18S* to give the relative gene expression. Each data point is the average of 3 biological replicates, and error bars represent the *SEM* between biological replicates. Results were analyzed using ANOVA, with confidence level of 95%, followed by a Tukey's post‐test. Same letter indicates no significant difference and different letters indicate significant difference between samples with *p* < .05

The extracted RNA was found to be pure and undegraded, and furthermore, changes to the transcriptome were dynamic, with transcripts exhibiting different expression kinetics at 24 and 72 hr following the inoculation of tomato with *P. carotovorum* subsp. *carotovorum* (*Pcc*) (Table 1). The fold expression varied from relatively low (>2 fold) to high (>10 fold) compared with the basal levels of non‐treated cells (Figure 1a–m).

**Table 1:** Differential expression of genes selected for investigating the response of tomato to infection with *Pectobacterium carotovorum* subsp. *carotovorum*

Gene	Downregulated at 24 hr postinoculation?	Upregulated at 24 hr postinoculation?	Downregulated at 72 hr postinoculation?	Upregulated at 72 hr postinoculation?
Endo‐1,3‐beta‐glucanase (PR2)	Yes			Yes
Proteinase inhibitor (PR6)		Yes		Yes
Chitinase (PR3)		Yes		Yes
PR‐1a	Yes		Yes	
Cytochrome P450		Yes		Yes
Defensin2 (PR12)	Yes			Yes
MYB transcriptor factor		Yes		Yes
CC‐NBS‐LRR resistance protein		Yes		Yes
EREBP		Yes	Yes	
HQT		Yes		Yes
PAL	No change	No change	No change	
SAR1(GTPases)	No change	No change		Yes
SGT1	Yes			Yes

The gene expression analysis showed that at 24 hr following bacterial inoculation, the expression profiles of some genes, namely, *Myb*, *EREBP*, *CytP450*, *HQT*, *PR3*, *PR6,* and *CC‐NBS‐LRR* were significantly upregulated with a maximum expression of 14‐fold observed with *Myb*. The lower significant increases with two‐ to fivefold changes were observed on the expression of *CytP450* and *CC‐NBS‐LRR* (Figure 1d,i,j,l). Others genes such as *EREBP*, *HQT*, *PR6,* and *PR3*, exhibited an increase with a similar fold change above fivefold compared with the expression of the control sample. A downregulation was observed for the expression of *SGT1*, *SAR1‐GTPase*, *PR1*, and *PR12*. *PAL* was not differentially expressed compared with the expression of the control sample.

At 72 hr following inoculation, the expression profiles of *Myb*, *SGT1*, *CytP450*, *SAR1‐GTPase*, *HQT*, *PR2*, *PR3*, *PR6*, *PR12*, and *CC‐NBS‐LRR* were significantly upregulated with a fold change varying between 2 and 11 compared with the expression of the control sample. *CytP450* and *PR6* exhibited the highest significant increase with a fold change >7 (Figure 1d,j). The other genes: *EREBP*, *PR1*, and PAL were downregulated compared with the expression of the control sample. These results point to the validity of using real‐time PCR to analyze the expression of host genes during plant–pathogen interactions. This study was not an exhaustive investigation of the genetic defense mechanisms of tomato against *Pcc* but a preliminary investigation to assess overall response to create a platform for future studies.

The selected genes investigated were previously reported to be involved in tomato (or related family) defense responses to pathogen attack (Alfano et al., 2007; Block, Schmelz, O'Donnell, Jones, & Klee, 2005; Djami‐Tchatchou et al., 2015; Hafez, Hashem, Balbaa, El‐Saadani, & Ahmed, 2013; Medeiros, Resende, Medeiros, Zhang, & Pare, 2009). Their expressions profiles detected by qPCR enable us to gain insight into the defense mechanism by which tomato responds to *Pcc*.

During plant–pathogen interactions, *EREBP* has been shown to be intimately related to defense responses, stress signaling pathways (Wang, Li, & Ecker, 2002); and the level of the biosynthesis of ethylene increases rapidly leading to the transcription of some defense‐related genes such as β‐1,3‐glucanase and chitinase class I (Sharma et al., 2010).

SGT1 regulates defense responses triggered by various pathogens, and it has been found from previous findings that *SGT1* is involved in plant resistance to pathogen attack (Djami‐Tchatchou et al., 2015; Meldau, Baldwin, & Wu, 2011). In this study, we found that the transcripts of *SGT1* exhibited an upregulation at 72 hr postinoculation. SGT1 was first found to confer resistance to *Peronospora parasitica* in Arabidopsis (Austin et al., 2002), and the *SGT1* gene interacts with *RAR1* (required for Mla12 resistance), to confer resistance by multiple *R* genes recognizing distinct avirulent *P. parasitica* or *Pseudomonas syringae* pv. *tomato* isolates (Muskett et al., 2002).

Plant *CytP450* has been shown to be involved in various biochemical pathways to produce primary and secondary metabolites such as phenylpropanoids, alkaloids, terpenoids, lipids, cyanogenic glycosides, and glucosinolates as well as plant hormones (Mizutani, 2012). *SAR1‐GTPase* is a small monomeric GTP‐binding protein belonging to the Rho subfamily which plays an important role in plant signal perception and transduction (Djami‐Tchatchou et al., 2015, 2017; Sanabria, Heerden, & Dubery, 2012). *SAR1‐GTPase* was not expressed at 24 hr, but was upregulated at 72 hr following *Pcc* inoculation.

The transcript of HQT, an important defense component (Mhlongo, Piater, Steenkamp, Madala, & Dubery, 2014; Niggeweg, Michael, & Martin, 2004; Yu & Jez, 2008), exhibited a rapid upregulation as early as 24 hr postinoculation, reached maximum levels then decreased but remained upregulated at 72 hr following *Pcc* infection. The patterns of expression of HQT are similar to the patterns of MYB transcription factor expression observed in this study (Figure 1a,f). A previous investigation reported that in potato, specific overexpression of an MYB transcription factor increased the level of CGA and the expression of HQT which resulted in CGA accumulation (Lepelley et al., 2007; Rommens et al., 2008). Our results indicate that HQT is involved in the response of tomato to *Pcc* infection with a positive regulation with MYB transcription factor. HQT is also known to be correlated with phenylalanine ammonia‐lyase (PAL), the first enzyme in the phenylpropanoid pathway linking primary metabolism to secondary metabolism (Tohge, Watanabe, Hoefgen, & Fernie, 2013). We found that the transcript of PAL was not differentially expressed at 24 hr with a slight nonsignificant downregulation at 72 hr. A positive correlation was expected between PAL and the HQT expression profile as PAL is involved in the biosynthesis of CGA. Our study showed that at 24 hr postinoculation, the transcripts of *CC‐NBS‐LRR* resistance and the pathogenicity‐related genes, PR3 and PR6, exhibited a significant upregulation as well as at 72 hr together with PR2 and PR12. Knowing that chitinases have lysozyme‐like activity, our results indicate that PR3 was induced at 24 and 72 hr postinoculation to directly inhibit *Pcc* as it was reported that chitinases with their lysozyme‐like activity may be directly inhibitory to many bacterial plant pathogens (Sudisha, Sharathchandra, Amruthesh, Kumar, & Shetty, 2012). A previous study showed that chitinase and β‐1,3‐glucanase were coordinately induced in infected leaf and flower tissue in response to pepper in the infection of *Xanthomonas campestris* pv*. vesicaroria* (O'Garro & Charlemange, 1994). Our results suggest that, PR6 was induced at 24 and 72 hr to inhibit the proteinase produced by *Pcc* in order to restrict *Pcc* spread in tomato. A similar observation was done in a previous study where it was found that PR6 was produced to restrict *P. syringae* spread in tomato (Koiwa, Bressan, & Hasigawa, 1997). Our results indicate that the upregulation of PR12 at 72 hr was to enhance tomato resistance to *Pcc*. The noninduction of PR1 observed in this study showed that it is not involved in the response of tomato to *Pcc*. In this study, genes of interest were selected based on the fact that they were previously reported to be involved in tomato (or related family) defense response to pathogen attack (Alfano et al., 2007; Block et al., 2005; Djami‐Tchatchou et al., 2015; Hafez et al., 2013; Medeiros et al., 2009). We conclude that *Pcc* infection of the tomato triggers the expression of a number of the genes selected, which is an indication of their involvement in defense. However, this preliminary finding requires further investigation such as the use of knockout tomato mutants to comprehensively assess gene function and the defense response.

## CONFLICT OF INTERESTS

The authors declare that they have no conflict of interest.

## AUTHOR CONTRIBUTIONS

ATD was involved in plant inoculation, sample collection, and performed the real‐time PCR and analyzed and interpreted gene expression data. LBT was involved in concept formulation and project administration. KN was involved in concept formulation, preparation of bacterial inoculum, plant inoculation, and sample collection. ATD, LBT, and KN were all involved in manuscript preparation each writing a section. Review and editing was done by all.

## ETHICS STATEMENT

None required.

## DATA AVAILABILITY STATEMENT

All data generated or analyzed during this study are included in this published article.

## APPENDIX 1

**Table A1:** Primers used in this study

GENES	Direction	Primer Sequence (5′−3′)	Accession number of target gene
Endo‐1,3‐beta‐glucanase (PR2)	Forward Reverse	ACAGCTCATACATGGCCTTCT ATTGGGCTTCTTGGTTGTGGTTGG	Medeiros et al. (2009)
Proteinase inhibitor (PR6)	Forward Reverse	CGGAGAATCTGAATGGGTAAGC GA ACAAGCCGTGGTAAAGGTCCACAA	Medeiros et al. (2009)
Chitinase (PR3)	Forward Reverse	ATGGCGGAAACTGTCCTAGTGGAA ACATGGTCTACCATCAGCTTGCCA	Medeiros et al. (2009)
PR−1a	Forward Reverse	GAGGGCAGCCGTGCAA CACATTTTTCCACCAACACATTG	Block et al. (2005)
Cytochrome P450	Forward Reverse	CTTAGTCGAGAATGGTAGTT AAACTCTCCAACTGTACTCT	Shi et al. (2013)
Defensin2 (PR12)	Forward Reverse	TCACCAAACTATTGGATTTCAA GACTCAATTTTTGACTTCTTAATCC	Hafez et al. (2013)
MYB transcriptor factor	Forward Reverse	CCTACCAATGATAGAA ATGGTACACACACCTACACG	Alfano et al. (2007)
Cc‐nbs‐lrr resistance protein	Forward Reverse	TCACCCAATGTTGAGGCTTG CTACCTCGTCGTGTTACATCTG	Shi et al. (2013)
EREBP	Forward Reverse	AGCATTTCCACCATCCTGTGTTGC TCCCAGATGAAGTTCTTGCAGATCCC	Djami‐Tchatchou et al. (2015)
HQT	Forward Reverse	GGAGGAGTATTCCACACTTTATC AGAGATGGAGGAGGATGATAC	Djami‐Tchatchou et al. (2015)
PAL	Forward Reverse	GTCACACATTACCACAATCAG GTGTTCCATAATAGCAGCAGCCT	Djami‐Tchatchou et al. (2015)
SAR1(GTPases)	Forward Reverse	ACCTAGACAAGAGAAACTATAGCCC TGGAGAAGGCTTCAGATGGATGTC	Djami‐Tchatchou et al. (2015)
SGT1	Forward Reverse	GAAGCTGGAGAACTACCTAATC AGGTTGACAGTTGGTTGAG	Djami‐Tchatchou et al. (2015)
18S ribosomal RNA gene	Forward Reverse	GGCAAATAGGAGCCAATGAA GGGGTGAACCAAAAGCTGTA	Djami‐Tchatchou et al. (2015)
Elongation factor α	Forward Reverse	ACC AGA TCA ATG AGC CCA AG AAG AGC TTC GTG GTG CAT CT	Djami‐Tchatchou et al. (2015)
